# Copper Isotopes and Copper to Zinc Ratio as Possible Biomarkers for Thyroid Cancer

**DOI:** 10.3389/fmed.2021.698167

**Published:** 2021-09-08

**Authors:** Latifa Sarra Kazi Tani, Alexandra T. Gourlan, Nouria Dennouni-Medjati, Philippe Telouk, Majda Dali-Sahi, Yahia Harek, Qian Sun, Julian Hackler, Moussa Belhadj, Lutz Schomburg, Laurent Charlet

**Affiliations:** ^1^Analytical Chemistry and Electrochemistry Laboratory, University of Abou Bekr Belkaid, Tlemcen, Algeria; ^2^ISTerre: Institut des Sciences de la Terre, Université Grenoble-Alpes, Université de Savoie Mont-Blanc, CNRS, IRD, IFSTTAR, Grenoble, France; ^3^Ecole Normale Superieure de Lyon, Centre National de la Recherche Scientifique, Université Claude Bernard Lyon 1, Lyon, France; ^4^Institute for Experimental Endocrinology, Charité-Universitätsmedizin Berlin, Corporate Member of Freie Universität Berlin, Humboldt-Universität zu Berlin, Berlin Institute of Health, Berlin, Germany

**Keywords:** copper, isotopes, zinc, biomarkers, thyroid cancer

## Abstract

Thyroid cancer is the most common endocrine cancer. There is no systematic screening for such cancer, and the current challenge is to find potential biomarkers to facilitate an early diagnosis. Copper (Cu) and zinc (Zn) are essential micronutrients involved in the proper functioning of the thyroid gland, and changes in their concentrations have been observed in the development of cancer. Previous studies have highlighted the potential ^65^Cu/^63^Cu ratio (δ^65^Cu) to be a cancer biomarker. This study tests its sensitivity on plasma samples (*n* = 46) of Algerian patients with papillary thyroid carcinoma and a set of corresponding biopsies (*n* = 11). The δ^65^Cu ratio in blood and tumor samples was determined using multi collector inductively coupled plasma-mass spectrometry (MC-ICP-MS), and their corresponding Cu and Zn plasma total concentrations using total reflection X-ray fluorescence (TXRF). Plasma concentrations of Cu were significantly higher (1346.1 ± 328.3 vs. 1060.5 ± 216.1 μg/L, *p* < 0.0001), and Zn significantly lower (942.1 ± 205.2 vs. 1027.9 ± 151.4 μg/L, *p* < 0.05) in thyroid cancer patients as compared to healthy controls (*n* = 50). Accordingly, the Cu/Zn ratio was significantly different between patients and controls (1.5 ± 0.4 vs. 1.0 ± 0.3, *p* < 0.0001). Furthermore, the δ^65^Cu plasma levels of patients were significantly lower than healthy controls (*p* < 0.0001), whereas thyroid tumor tissues presented high δ^65^Cu values. These results support the hypothesis that Cu isotopes and plasma trace elements may serve as suitable biomarkers of thyroid cancer diagnosis.

## Introduction

Thyroid cancer is the most frequent malignancy of the endocrine system, responsible for almost 90% of endocrine cancers (1, 2), and accounting for the Surveillance, Epidemiology, and End Results (SEER) Stat Fact 4% of all new cancer cases in the US (3). Its incidence is increasing with 567.000 new cases annually, ranking it the 9th place worldwide. It is diagnosed three times more in woman (10.2 per 100,000) than in men (3.1 per 100,000) (4). In Algeria, thyroid cancer incidence is increasing, taking now the 3rd place in women after breast and colorectal cancer, while in 2006 it occupied the 5th position in frequency (5, 6). Four types of thyroid cancer are histologically distinguished. The most common is papillary thyroid carcinoma (PTC) representing 80 to 90% of new thyroid cancer cases (7). There is no systematic detection of thyroid cancer, and the diagnosis is made incidentally in 25% of cases during the follow-up of another thyroid disease (2, 8). Therefore, a current challenge lies in identifying suitable biomarkers for supporting a fast diagnosis of thyroid cancer.

The link between metal trace elements and cancer has not been yet proofed nor much studied, especially in Algeria. Copper (Cu) is an essential metallic trace element for living organisms (9). Changes in Cu metabolism have been observed concomitantly with the development of cancer (10). However, Cu is not a carcinogen, but an increased Cu bioavailability may lead to an increased production of ATP, which is used by cancer cells during proliferation (10). Thus, Cu contributes to the proliferation of transformed cells by providing the energy required for cell cycle progression (11). As Cu has a short residence time in the bulk human body (<6 weeks), it is a potential indicator for rapidly evolving diseases such as cancers (12). Like Zinc (Zn), Cu is a catalytic cofactor in Cu/Zn superoxide dismutase I enzyme (SOD1), and it is the interaction between Cu and Zn that allows the enzyme to function properly (13). This enzyme has a very important role in antioxidant defense system of cells, as it converts superoxide into oxygen and hydrogen peroxide. A disturbance of its expression is associated with various cancers such as hepatocellular carcinoma (14), and overexpression of SOD1 promotes tumor growth in lung cancer cells and reduce apoptosis (15). Although significantly elevated concentrations of Cu and Cu/Zn ratio have been observed in the plasma of patients with breast cancer (16, 17), it seems that trace metal imbalances (Cu/Zn) are clinically more sensitive indices of disease than the concentration of any single trace metal alone (13).

Copper naturally has two stable isotopes: ^63^Cu (69.2%) and ^65^Cu (30.8%) (18). The isotopic abundances of copper in human serum can vary according to sex, menopause, and metabolic diseases (19, 20). The conventional delta value (δ ^65^Cu) is used to report the Cu isotope abundances and corresponds to the relative deviation of ^65^Cu/^63^Cu ratios in the measured samples from its value in reference material NIST SRM 976. Some studies observed variations in δ^65^Cu and indicated an altered metabolism of cancer cells compared to normal cells (12, 21). The differences (corresponding to the isotopic fractionation) are still poorly understood. Several steps such as Cu reduction, Cu transport across the membrane or Cu binding to organic ligands have the potential to generate fractionation and different metabolic processes such as hypoxia and angiogenesis (22). The *ab initio* calculations can predict quantitively the variation of Cu isotope abundance, and can provide an overview into a biological process in biological reactions: in tumor, high δ^65^Cu values represent a reserve of the circulating ^63^Cu isotope, due to absorption by tumor cells, conversely low values indicating an enrichment of the lighter ^63^Cu isotope in the serum (12, 21). Recent studies on patients with different types of cancer demonstrate that Cu present in the blood is enriched in light isotopes relative to healthy controls (12, 21, 23). Consequently, Cu isotopes have strong potential to constitute meaningful markers of cancer detection. For the moment, this type of studies was only applied for breast, ovarian, and colorectal cancer (12, 21, 23). In this study, its suitability for thyroid cancer is tested by comparing the abundance of ^63^Cu and ^65^Cu in plasma and tissue biopsies of thyroid cancer patients, along with an assessment of total plasma Cu and Zn concentrations. Our results support the notion that Cu isotopes and total trace elements may support early thyroid cancer detection and diagnosis.

## Materials and Methods

### Blood and Tissues Samples

The recruitment of patients was done at University Hospital Centers of TLEMCEN and ORAN (Western Algeria) from June 2018 to May 2019. Forty-six patients with PTC and fifty control subjects were randomly and consecutively selected for the study without applying specific exclusion criteria. All participants had been informed of the purpose of this study, their informed consent had been requested and signed in advance, and people refusing to participate were excluded from the study.

The blood samples, before ablation, of the participants were collected using a venipuncture into 4 mL heparinized tubes and centrifuged at 1,100x g (relative centrifugal force) for 10 min using a Sigma 2-16P centrifuge, the plasma was recovered in aliquots of 500 μl. A total number of 11 investigated tissues with thyroid cancer were collected after surgery (thyroidectomy). A diagnosis of thyroid cancer disease was confirmed by an independent pathologist after postoperative pathohistological analysis of thyroid tissues. Plasma of the participants and thyroid tissues samples were stored and kept on −80°C freezer until further manipulation.

A questionnaire was filled, via a face-to-face interview, with every participant considering the anthropometric and socio-cultural parameters. Patients presenting other types of malignancies, chronic diseases, or taking a chronic drug therapy were excluded. This study was conducted in accordance with Algerian law (25/2006, resolution No. 387) and approved by the Scientific Ethics Deontology Committee of Tlemcen University.

### Copper and Zinc Status Assessment

Total plasma Cu and Zn concentrations were determined by total reflection X-ray fluorescence (TXRF) using a benchtop analyzer (S2 Picofox, Bruker nano GmbH, Berlin, Germany), essentially as described (24, 25). All samples were supplemented with a gallium standard for calibration. Aliquots were applied to cleaned and polished quartz glass slides and dried, before being analyzed by X-ray fluorescence. A seronorm serum standard (Sero AS, Billingstad, Norway) served as control and was included in all assay runs. The concentrations determined for total Cu and Zn were within the specified range of the standard, and the inter-assay coefficient of variation (CV) was below 15% during the analysis.

### Copper Isotope Assessment

Plasma (200 μl) and thyroid tumor biopsies (200 μg) were mineralized on a hot plate in a mixture of nitric acid and hydrogen peroxide. Cu was isolated from the other elements using quartz columns following the protocol described in (26, 27). The δ ^65^Cu were determined using a Nu Plasma MC-ICP-MS (Nu Instruments, Wrexham, UK) of ENS-Lyon. As MC-ICP-MS suffers from a stable bias, a correction was systematically applied for each isotopic ratio using a constant and known standard of Zn which was added for each sample and standard. Typical external reproducibility on δ ^65^Cu determined from multiple replicates of samples is ~ 0.05‰ for each session. The conventional delta values, δ ^65^Cu, measured for each sample is defined by the formula below:

$$\delta^{65}Cu=\left[\frac{\left({}^{65}Cu{/}^{63}Cu\right)sample}{\left({}^{65}Cu{/}^{63}Cu\right)IStd}-1\right]\times 10\;000$$

Procedural blanks were below 0.06 ng on average and can be neglected regarding the quantities of Cu isolated.

### Statistical Analysis

Normality of the data was tested using Kolmogorov–Smirnov test. All analysis (Chi-square test, two-samples *t-*test, Mann–Whitney U, Kruskal–Wallis, Spearman correlation, linear regression, and Receiver Operating Characteristics “ROC”) were performed using IBM SPSS Statistic software version 23 (IBM Corporation, USA). The results of continuous variables are provided in mean (95% K & Sr peaks CI) and median (IQR) and of categorical variables in percentages (%). When the *P*-value was < 0.05 the result was considered as statistically significant, and the degree of the differences are marked as: ^*^p < 0.05, ^**^p < 0.01, ^***^p < 0.001, and ^****^p < 0.0001. All figures were created with GraphPad Prism 9.0.1.

## Results

The study population included 96 individuals, 46 cases with PTC and 50 healthy controls from western Algeria. This population was predominantly female with a ratio of 9:1, which reflects the difference between the sexes in the prevalence of thyroid cancer (28). The average age of the group was 42 ± 13 years. The characteristics of the study population are described in Table 1. No significant differences have been observed between cases and controls in the anthropometric parameters. The thyroid parameters were compared with international standards. A value was considered as abnormal when it was outside the standard range: Thyroid Stimulating Hormone (TSH) 0.3 < TSH <5.0 μIU/mL, anti-thyroid peroxidase antibodies (TPO-ab) <34 UI/mL and thyroglobulin (TG) levels 3.0 < TG <40 ng/mL. We observed that thyroid biomarkers are highly perturbed in the individuals with thyroid cancer compared to the international standard.

**Table 1:** Characteristics of the study population.

**Variables**	**PTC**	**Healthy**	***P*-value**
	**patients**	**controls**	
**Number of participants**	46	50	
**Number of biopsy tissues**	11	0	
**Sex (%)**			0.19
Women	91%	85%	
Men	09%	15%	
**Age (years)**			0.27
Median (IQR)	39 (29, 50)	42 (36, 48)	
Mean (95% CI)	40 (36, 49)	43 (39, 46)	
			0.19
>50 years (%)	28 %	20 %	
<50 years (%)	72 %	80 %	
**BMI (kg/m** ^2^ **)**			0.09
Median (IQR)	27.8 (23.3, 32.2)	25.9 (23.2, 30.5)	
Mean (95% CI)	28.3 (26.6, 30.0)	26.4 (25.2, 27.7)	
**Smoking (%)**			0,27
Yes	09%	14%	
No	91%	86%	
**TSH (μIU/mL)**		/	
Median (IQR)	92.4 (70.9, 101.0)		
Mean (95% CI)	82.9 (75.6, 90.0)		
**TPO-ab (Ul/mL)**		/	
Median (IQR)	16.4 (14.7, 21.0)		
Mean (95% CI)	38.7 (15.5, 21.9)		
**TG (ng/mL)**		/	
Median (IQR)	11.9 (4.7, 87.9)		
Mean (95% CI)	109.5 (32.8, 186.24)		

*BMI, Body Mass Index; TSH, Thyroid Stimulating Hormone; TPO-ab/ anti-Thyroid Peroxidase antibodies; TG, Thyroglobulin*.

**Figure 1:**
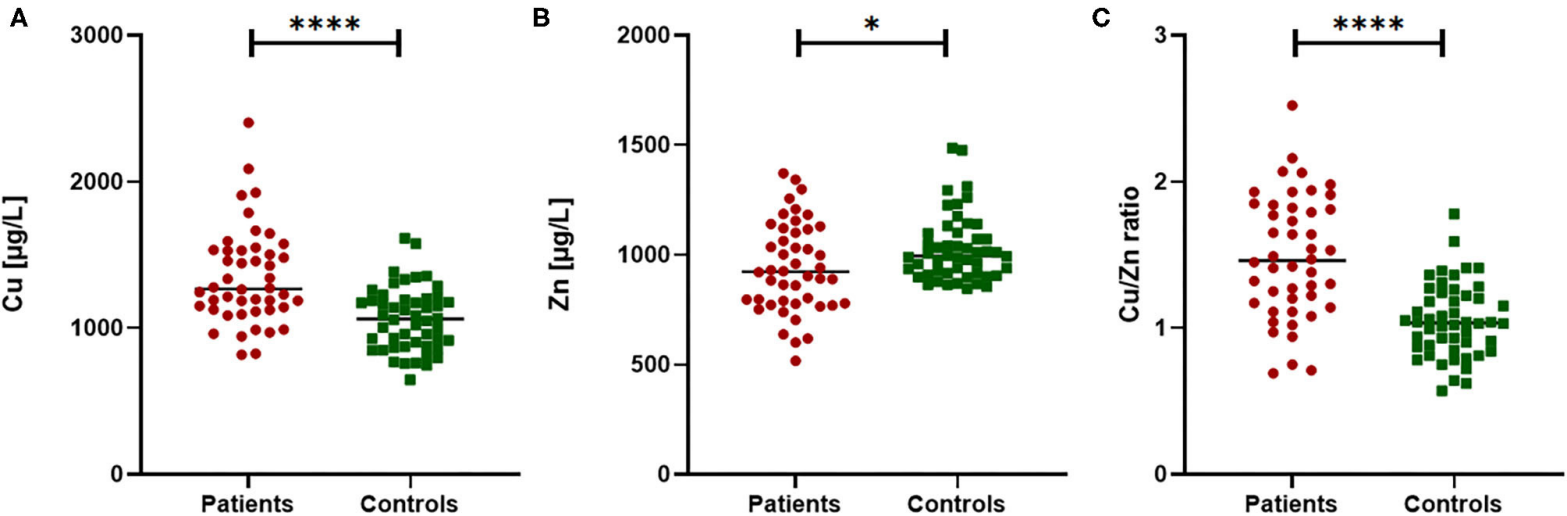
Comparison of Cu, Zn and Cu/Zn ratio in PTC patients vs. healthy controls. Significant differences were observed in plasma Cu concentration **(A)**, Zn concentration **(B)** and in the Cu/Zn ratio **(C)**. Mann Whitney test for non-normal distribution or *T*-test for variables normally distributed; *p < 0.05 and ****p < 0.0001.

The trace element status of Cu and Zn as well as the Cu/Zn ratio were compared between PTC patients and healthy controls (Figure 1). Significant higher differences were observed in plasma of Cu levels and Cu/Zn ratio between cases and controls (*P* < 0.0001). The average Cu levels was 1346.1 ± 328.3 μg/L in PTC patients and 1060.5 ± 216.1 μg/L in controls. Conversely, significant lower Zn plasma levels were observed (*P* < 0.05) between the two groups, with average 942.1 ± 205.2 μg/L and 1027.9 ± 151.4 μg/L in PTC and healthy subjects, respectively.

We also evaluated, using Spearman's correlation test, the association between trace metals (Cu and Zn) and thyroid parameters (TSH, TPO-ab, TG). However, no significant associations were observed in PTC patients (*P* > 0.05).

Copper and Zn concentrations were classified into 3 classes according to international reference values (Table 2). The Cu value (median 1,060 μg/L) of our control group was close to the corresponding reference ranges from other countries, such as Germany (1,020 μg/L, with a range of 804–1,620 μg/L) (29). In comparison to the healthy controls, more than 16% of patients with PTC had elevated Cu concentrations. Cu levels in the majority of the studied population was within the reference range. In our patients, Zn concentrations were lower than in our controls, but most of the values were still within the reference range [785–1,046] μg/L (28, 30). However, the distribution of Cu and Zn concentrations in the classes of the two groups remained significantly different (*P* < 0.0001).

**Table 2:** Prevalence of elevated Cu and deficient Zn concentrations in PTC patients and healthy controls in comparison to international reference ranges.

**Reference values**	**PTC**	**Healthy**	***P*-value**	**Reference**
	**patients (%)**	**controls (%)**		
**Cu [μg/L]**			0.0001	(29)
n % (Cu ≤ 804 μg/L)	0	14		
n % (804 < Cu <1,620 μg/L)	84	86		
n % (Cu ≥ 1,620 μg/L)	16	0		
**Zn [μg/L]**			0.0001	(30)
n % (Zn ≤ 785 μg/L)	26	0		
n % (785 < Cu <1,046 μg/L)	46	68		
n % (Cu ≥ 1,046 μg/L)	28	32		

**Figure 2:**
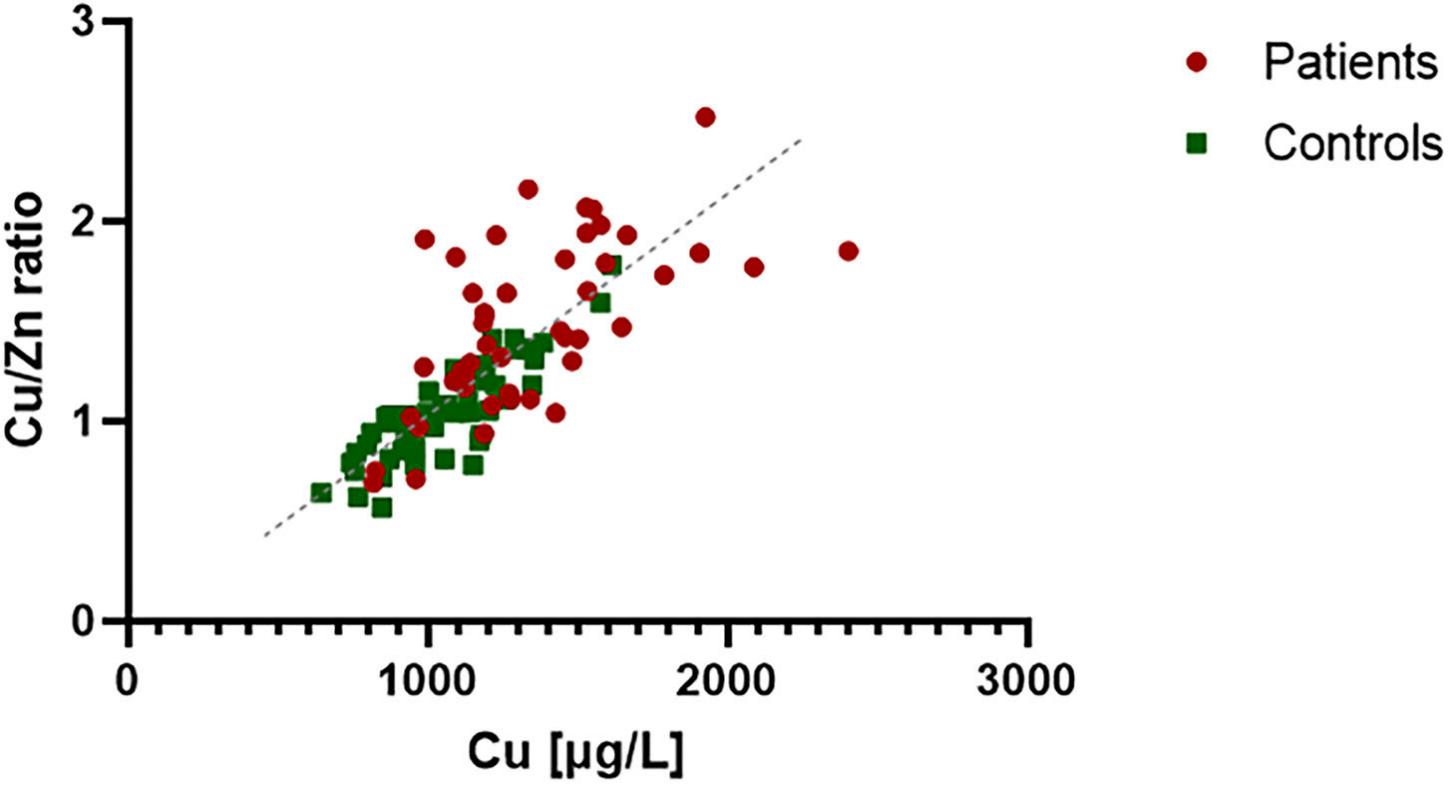
Correlation analysis between Cu and Cu/Zn ratio in plasma of PTC patients and healthy controls. The control group expressed a strong correlation (*R*^2^ = 0.85), which reflect Zn regulation in the body (x/y = Zn). Zn concentration in PTC plasma seems to be less regulated and variable compared to controls (*R*^2^ = 0.39).

The correlation between Cu and Cu/Zn ratio in the PTC patients (in red, in Figure 2) and in the healthy controls (in green, in Figure 2) was studied. They were widely used as an indicator in breast cancer (12). In the control group, we observed a clear correlation reflecting a strict regulation of Zn levels in the body (x/y = Zn) compared to the cases (*p* = 0.001). The plasma of PTC showed a high degree of deregulation in Cu and Zn.

**Figure 3:**
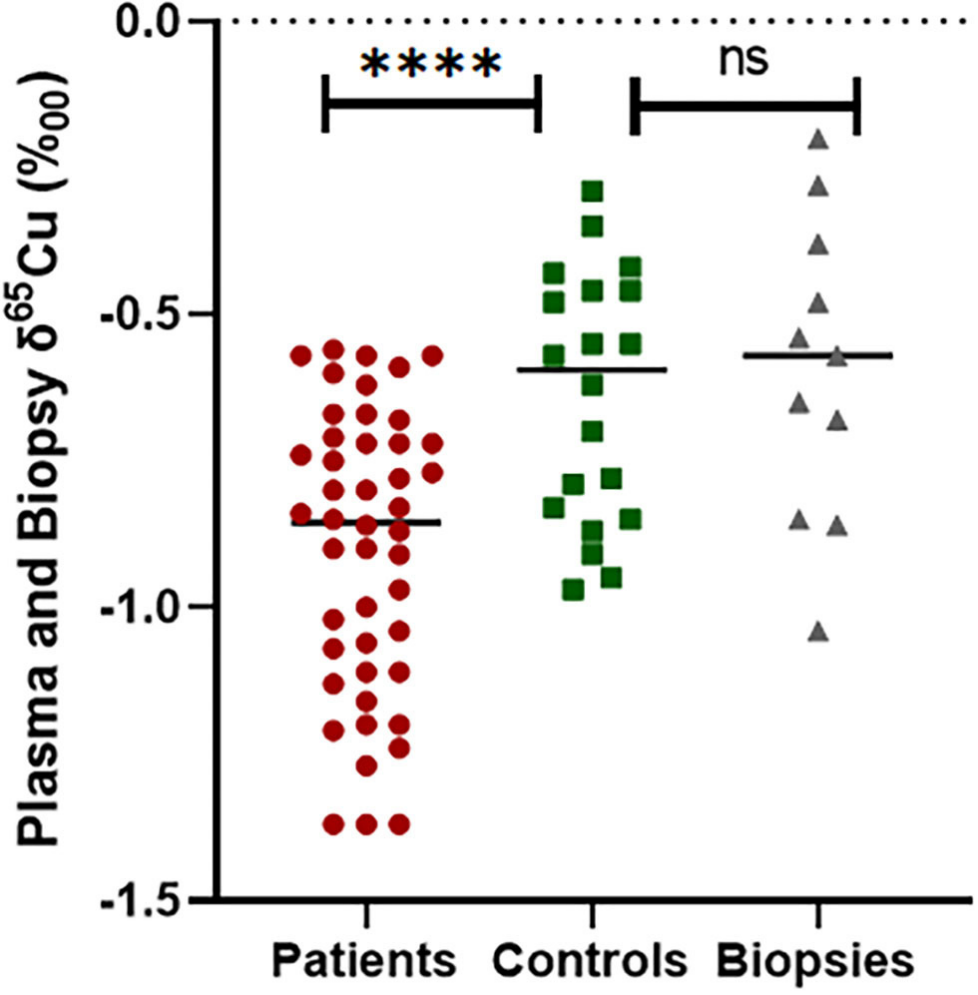
Comparison of δ^65^Cu values between healthy controls plasma and thyroid cancer patients' plasma and biopsy, ****p ≤ 0.0001 and ns, no significant differences.

We treated each δ^65^Cu plasma measurement for each patient, tissue, and control as an independent measurement (Figure 3). PTC plasma samples had a Cu isotope ratio ranking from −1.38 to −0.56‰ (median −0.86‰, mean −0.90 ± 0.24‰) and remained significantly lower (*p* < 0.0001) compared to the healthy controls (between −0.2 and −0.9 ‰, median −0.60‰, mean −0.61 ± 0.21‰).

Nevertheless, there was no significant difference between the δ^65^Cu measured in controls and the δ^65^Cu measured in the thyroid tissue biopsies (*p* = 0.163). We then compared the mean δ^65^Cu of patients and thyroid biopsies and found that there was a significant difference as a mirror effect (*p* < 0.05). We also compared plasma δ^65^Cu values with age, sex and thyroid parameters but no association was found in PTC patients (*p* > 0.05).

**Figure 4:**
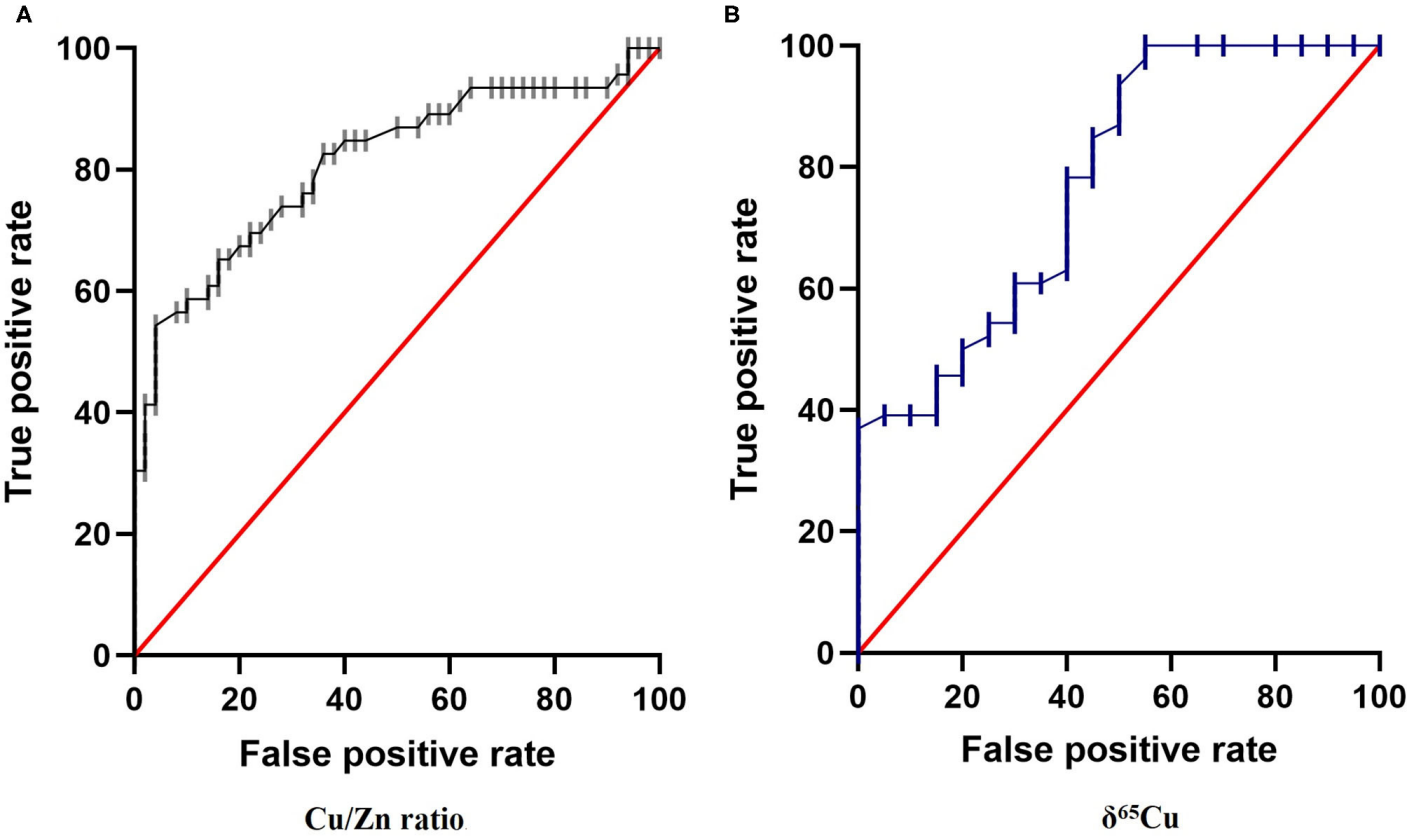
Receiver Operating Characteristics (ROC) curve for: **(A)** Cu/Zn Ratio and **(B)** δ^65^Cu in the plasma of papillary thyroid carcinoma. We place the True Positive decision on the ordinate axis and False Positive rate on the abscissa to form the ROC diagram. The area under the ROC curve (AUC) obtained for the present data were 0.81 and 0.78, respectively (*p* < 0.001) supports the hypothesis that Cu/Zn ratio and δ^65^Cu have a strong potential to be a novels biomarker in diagnosis for thyroid cancer.

Finally, we analyzed Cu/Zn ratio and δ^65^Cu data with Receiver Operating Characteristics (ROC) curve analysis (Figure 4). The ROC curve allows us to evaluate the probability that a result obtained is a true positive or a false positive, and the area under the curve (AUC) indicates the probability to get a positive value in front of a negative one. AUC vary between 0.5 (pure chance) and 1.0 (totally reliable). In our case, we evaluated the use of Cu/Zn ratio and δ^65^Cu as novel biomarkers in diagnostic tool for thyroid cancer. The results indicate an acceptable degree of accurate diagnosis and prediction, with an AUC = 0.81 for Cu/Zn axis (*p* < 0.0001) and an AUC = 0.78 for δ^65^Cu of axis (*P* < 0.0001), respectively, to discriminate between controls and cancer patients (Figure 4).

## Discussion

In this study, we present a comparison of trace elements in Algerian healthy subjects and patients with a diagnosis of PTC. Our results indicate that both total plasma Cu and Zn concentrations with the Cu/Zn ratio as well as the δ^65^Cu marker yield fast, low-cost and meaningful insights and may support a thyroid cancer diagnosis. However, the nature of our study is explorative, and a single sample per patient only was available for analysis. For these reasons, the data need to be interpreted with the due caution until the results have been verified in larger study cohorts. Nevertheless, the measurements were conducted by scientists blinded to the clinical phenotypes, and the study groups were of sufficient size to support further research on these novel biomarkers of thyroid cancer.

### Metals Concentrations and Ratios

We observed that blood Cu concentrations were significantly higher and Zn concentrations significantly lower in thyroid cancer patients compared to healthy controls. Cu and Zn are important for thyroid gland function, in metabolism and in synthesis of thyroid hormones (31). Such changes in concentration of trace metal can affect the balance between oxidant and antioxidant in the body and thus the endocrine system and can result in different thyroid diseases such as hyperthyroidism, hypothyroidism, Hashimoto's disease, and cancer (31, 32).

First, Copper interact with tyrosine amino acid metabolism which is necessary for production of thyroid hormones (33). Cu regulates the excessive absorption of thyroxine (T4) by monitoring the calcium levels and can eliminate free radicals and reduce some damage caused in the cells during the synthesis of thyroid hormones (34). It is reported in some studies, that high levels of Cu may cause an oxidative stress that can alter normal thyroid function (31, 33, 35, 36). Some studies reported high Cu blood levels in cancer, and they correlated with grade and therapies response (10, 37). Ishida et al. (11) using a mouse model genetically modified of human cervical carcinoma reported a high expression of Cu transporter Ctr1 in the cancer cells, and thus tumors might have a higher dependence for Cu. Cu has the particularity to act as antioxidant and pro-oxidant (34, 38). Cu is considered as switch activating the angiogenesis process of tumoral cells. Abnormally high concentration of Cu can facilitate the proliferation of tumors by damaging DNA with toxic free hydroxyl radicals. In addition, high serum Cu concentrations are correlated with patients having different types of cancer and in most cancers, it is considered as hallmark of cancer cells (39). But for the moment, the effect of Cu mechanism in angiogenesis is still unclear (10). Cu is actively involved in the process of tumor progression, particularly during angiogenesis and metastasis (40). Clinical studies in rodent models show that Cu supplementation significantly increases tumor growth in breast, pancreatic and lung cancer (41). Cu regulates the expression and the secretion of certain angiogenic factors, such as vascular endothelial growth factor (VEGF), fibroblast growth factors (FGF) and interleukin-1alpha (IL-1α). Therefore, Cu indirectly participates in angiogenesis and tumor nutrition (42).

In addition, during the inflammatory response, Cu is mobilized and accumulates in inflamed tissues, contributing to the defense against various infections. However, chronic inflammation is a risk factor in the development of cancer. Certain inflammatory cytokines, such as IL-6 and IL-17, are diagnostic markers for early phase of inflammation and they interact in the promotion of tumors and cancer progression and they act in the regulation of Cu uptake mechanisms, thus contributing to the accumulation of Cu in cancer cells (24, 43).

Secondly, Zinc is a micronutrient required for the biosynthesis of thyroid hormones, and Zn affects the activity of 5′-deiodinase for the conversion of T4 to triiodothyronine (T3) (44, 45). In addition, Zn is necessary for the synthesis of TSH from pituitary and TRH from hypothalamus (46, 47). Kralik et al. (48) showed Zn to be required for the correct metabolism of thyroid hormone, and Zn deficiency might have a negative effect on the activity of normal thyroid. Meta-analysis conducted by Gumulec et al. (49) provided evidence for Zn level to alter cancer-specific tissue, with low Zn concentration associated with most tumors and in particular with thyroid carcinoma.

Few studies reported a lower levels of Zn and relatively higher levels of Cu in thyroid cancer patients (46). The study of Lin et al. (50) found significantly lower concentration of Zn (0.73 vs. 1.01 mg/L) with significantly higher levels of Cu (1.16 mg/L vs. 0.92 mg/L) in patients with hepatocellular carcinoma compared to controls. Baltaci et al. (46) found a negative correlation between lower levels of Zn and higher levels of Cu in the same patients. There is an antagonistic relationship between Cu and Zn; a diet rich in zinc (overdose) may lead to disruption of Cu absorption, and conversely a high dose of Cu may decrease Zn absorption (51, 52). This could explain the results obtained in our patients showing a perturbation in their Cu/Zn ratio. Alternatively, the altered Cu/Zn ratio may reflect the ongoing inflammation during thyroid cancer, which may set in early. In general, Cu is upregulated as positive acute phase reactant, whereas Zn tends to decline in response to inflammation. This inverse response to cytokines may contribute and synergize with the antagonistic changes of trace element absorption mentioned above.

Furthermore, during the synthesis of thyroid hormones, free radicals (ROS) are produced (53). It has been reported that SOD enzymes are involved in the thyroid gland functioning (54). Through cellular protection mechanisms, SOD enzymes eliminate excess ROS and prevent tumor progression through cellular protection mechanisms. On the other hand, the disturbance of the Cu-Zn homeostasis and an overproduction of ROS can lead to DNA damage, protein modification and eventually to the development of cancer (10). This may also explain the Cu/Zn imbalance observed in our thyroid cancer patient cohorts.

We found higher Cu/Zn ratio in PTC patients compared to healthy controls, this result was similar to previous studies conducted on breast, prostate, colorectal, and uterine cervical cancer (55, 56). The Cu/Zn ratio is considered as a potential biomarker for cancer detection (24). Our results support its potential value for PTC detection, thereby expanding its potential use as screening and diagnosis tool to the thyroid gland.

Some studies reported the influence of thyroid hormones in Cu and Zn metabolism (36, 57). It was observed that thyroid hormones can influence the expression of Cu transport proteins (ATP7A and ATP7B) and regulate serum Cu levels by controlling the production of the Cu-transport protein ceruloplasmin (58). In addition, it was found a significant correlation between TSH and Zn levels in hyperthyroidism (59). However, in our study we found no significant association between trace metals and thyroid parameters.

### Copper Isotopic Composition

With the development of new sensitive techniques such as MC-ICP-MS a search for non-invasive low-cost biomarkers has emerged, aiming at the development of early disease diagnosis. In this study, the δ^65^Cu marker is used and based on the determination of two stable Cu isotopes concentration ratio (^65^Cu/^63^Cu). We evaluated the Cu fractionation in blood and in tissues samples of patients with PTC and found significantly lower plasma value of δ^65^Cu in patients with PTC compared to healthy controls, and in a mirror image, significant higher values of δ^65^Cu in thyroid tumor tissue. Studies conducted in breast, colorectal and ovarian cancer found similar results, and have shown lighter enrichment in ^63^Cu isotope in serum and heavier enrichment in the ^65^Cu isotope in tissue, due to a preferential uptake of the heavy isotope by tumor cells (12, 21).

We used ROC curve analysis (Figure 4B) to evaluate the use of Cu isotope as diagnostic biomarker for thyroid cancer. The ROC curve is a widely used tool in diagnostic medicine. It represents the ability of a test to discriminate between the patient and non-patient population (60). The ROC curve represents on the ordinate the proportion of positive tests among the patient's population (the sensitivity), in our case the δ^65^Cu values of the thyroid cancer patients, vs. the proportion of positive tests among the non-diseased population. The reliability of the results is assessed by the AUC which ranges from 0.5 (pure chance) to 1.0 (totally reliable test). The ROC curve represents all the points calculated for each δ^65^Cu sample value. In the present case of thyroid cancer diagnosis, the AUC is equal to 0.78 and were similar to that observed in breast cancer (12). The use of δ^65^Cu as a potential diagnostic tool for thyroid cancer seems very promising.

The isotopic variability can be influenced by different mechanisms such menopause (61, 62) or biological sex (20). δ^65^Cu values seem also to vary according to diet. In fact, Jaouen et al. (20) demonstrated that Cu isotope composition of healthy Yakut's serum are significantly different (lighter) than observed in reference panel of Japanese and European population and supposed to be related to the isotope composition of the diet. The isotopic fractionation in diet can be generated during the intestinal absorption or excretion of food inducing differences in the isotopic composition of the human body or reflect different biogeochemical origins of the food items. Furthermore, it has been observed that metabolic reactions can also cause isotopic fractionation. In several diseases, such as cancer, this metabolic change can be measured (63).

As previously highlighted, the cause of isotopic fractionation in the case of cancer is not well understood but it has been suggested by Telouk et al. (12), that the hypoxic environment in tumor cells would be at the origin of the decrease of δ^65^Cu in the serum and the enrichment of δ^65^Cu in the tissues. It has been reported that in solid tumors there is a preferential chelation of the heavy ^65^Cu isotope in the tumor tissues and the release of the light ^63^Cu isotope into the blood (12, 19). However, some studies on hematological cancer found different δ^65^Cu values in tumor patients vs. controls, but at the same time similar isotopic fractionation in plasma and solid tumors, suggesting that the mechanism responsible for this distribution was not related to preferential absorption and release from the tumor but was caused by the proliferation of tumor cells (23). The mechanism of isotopic fractionation is thus not yet fully understood, and further studies in cellular and subcellular range are needed to elucidate this variation.

## Conclusion

The assessment of Cu and Zn plasma levels showed higher Cu and lower Zn concentrations in PTC patients compared to healthy controls, yielding a highly significant difference in the plasma Cu/Zn ratio. In addition, the Cu isotopic composition was strongly different in the thyroid cancer, patients as compared to healthy controls. Notably, the δ^65^Cu values differed in opposite directions in plasma vs. the thyroid tumor tissue samples. Our study supports the hypothesis that trace element concentrations and isotopic composition have a high potential to be used as additional biomarkers in the detection of thyroid cancer. Further molecular studies are needed for a better comprehension of the mechanisms involved in these processes.

## Data Availability Statement

The original contributions presented in the study are included in the article, further inquiries can be directed to the corresponding author/s.

## Ethics Statement

The studies involving human participants were reviewed and approved by Scientific Ethics Deontology Committee of Tlemcen University. The patients/participants provided their written informed consent to participate in this study.

## Author Contributions

AG, LK, LC, and ND-M designed the study. MB, LK, and MD-S collected the sample and data via face-to-face interviews. LK, AG, and PT performed copper isotope assessment. LS, QS, and JH did the Cu and Zn measurements. LK and YH performed the statistical analyses. LK, AG, and ND-M prepared the manuscript. LC and LS corrected the final version. All authors have read and agreed to the published version of the manuscript.

## Funding

This study benefited from the financial support from ISTerre and the inter-Instituts of CNRS. Deutsche Forschungsgemeinschaft (DFG), Research Unit FOR-2558 (Scho 849/6-2) and CRC/TR 296 (LocoTact, P17) to LS.

## Conflict of Interest

The authors declare that the research was conducted in the absence of any commercial or financial relationships that could be construed as a potential conflict of interest.

## Publisher's Note

All claims expressed in this article are solely those of the authors and do not necessarily represent those of their affiliated organizations, or those of the publisher, the editors and the reviewers. Any product that may be evaluated in this article, or claim that may be made by its manufacturer, is not guaranteed or endorsed by the publisher.
